# Machine Learning Algorithms for Detection and Classifications of Emotions in Contact Center Applications

**DOI:** 10.3390/s22145311

**Published:** 2022-07-15

**Authors:** Mirosław Płaza, Sławomir Trusz, Justyna Kęczkowska, Ewa Boksa, Sebastian Sadowski, Zbigniew Koruba

**Affiliations:** 1 Faculty of Electrical Engineering, Automatic Control and Computer Science, Kielce University of Technology, Al. Tysiąclecia P.P. 7, 25-314 Kielce, Poland; j.keczkowska@tu.kielce.pl; 2 Institute of Educational Sciences, Pedagogical University in Kraków, ul. 4 Ingardena, 30-060 Cracow, Poland; slawomir.trusz@up.krakow.pl; 3 Faculty of Humanities, Jan Kochanowski University, ul. Żeromskiego 5, 25-369 Kielce, Poland; ewa.boksa@wp.pl; 4 DHL Parcel Poland, ul. Osmańska 2, 02-823 Warszawa, Poland; s-sadowski@wp.pl; 5 Faculty of Mechatronics and Mechanical Engineering, Kielce University of Technology, Al. Tysiąclecia P.P. 7, 25-314 Kielce, Poland; ksmzko@tu.kielce.pl

**Keywords:** call/contact center, emotions recognition, virtual assistant, voicebot, chatbot

## Abstract

Over the past few years, virtual assistant solutions used in Contact Center systems are gaining popularity. One of the main tasks of the virtual assistant is to recognize the intentions of the customer. It is important to note that quite often the actual intention expressed in a conversation is also directly influenced by the emotions that accompany that conversation. Unfortunately, scientific literature has not identified what specific types of emotions in Contact Center applications are relevant to the activities they perform. Therefore, the main objective of this work was to develop an *Emotion Classification for Machine Detection of Affect-Tinged Conversational Contents* dedicated directly to the Contact Center industry. In the conducted study, Contact Center voice and text channels were considered, taking into account the following families of emotions: anger, fear, happiness, sadness vs. affective neutrality of the statements. The obtained results confirmed the usefulness of the proposed classification—for the voice channel, the highest efficiency was obtained using the Convolutional Neural Network (accuracy, 67.5%; precision, 80.3; F1-Score, 74.5%), while for the text channel, the Support Vector Machine algorithm proved to be the most efficient (accuracy, 65.9%; precision, 58.5; F1-Score, 61.7%).

## 1. Introduction

In recent years, the study of affect from data generated in a digital environment has become one of the major tasks of the field of Affective Computing [1]. This approach, through the development of automated information systems that recognize affective states, attempts to bridge the communication gap that exists between the emotional state of a human (user) and the programmed behavior of an information system (device). Activities undertaken in this area are aimed at creating the most universal and optimal models of representation of affective states in computer information systems for use in specific branches of the economy.

The rapid development and increasingly widespread use of artificial intelligence technologies has led to a significant increase in interest and development of systems that automatically recognize affective states. These systems are designed to recognize, accurately interpret, and respond to the recognized emotional states of the communicating individual [2,3,4,5,6]. There is an entire class of solutions called multimodal-based affective human–computer interaction that enables computer systems to recognize specific affective states. Emotions in these approaches can be recognized in many ways, including those based on:

▪ Voice parameters (timbre, raised voice, speaking rate, linguistic analysis and errors made) [7,8,9,10];
▪ Characteristics of writing [11,12,13,14,15];
▪ Changes in facial expressions in specific areas of the face [16,17,18,19,20];
▪ Gestures and posture analysis [21,22,23,24];
▪ Characterization of biological signals, including but not limited to respiration, skin conductance, blood pressure, brain imaging, and brain bioelectrical signals [25,26,27,28,29,30];
▪ Context—assessing the fit between the emotion and the context of expression [31].

Solutions combining all or selected elements from among the above are known to form multimodal communication systems [32,33]. Information about affective states recognized automatically by information systems can be used in a variety of industries, including the following:

▪ Marketing, for creating personalized advertisements based on a customer’s emotional state; monitoring customer satisfaction levels [34,35,36,37];
▪ Robotics, for designing service robots that detect and respond to emotions [38,39,40,41];
▪ Education processes, for improving learning effectiveness [8,42,43,44];
▪ In the industry, by creating personalized offerings for specific audiences [45].

Table 1 summarizes selected solutions of systems that use information about recognized human affective states in their functionalities.

Accurate identification of human emotions by a computer system is a difficult task, determined by many factors, including the specificity of language corpora and cultural differences in the population [48,49,50]. An additional source of limitations is the available technological solutions, including algorithms for identifying affective coloring of behavior, as well as the specificity of the available material, which impacts the quality of the processed signals. Finally, the expression of emotions is influenced by individual differences, their socialization and educational experiences, and the situational context, which together result in the same emotional states expressed in various ways by different people [51,52]. Affective computing, in some approaches, indeed simplifies the problem by assuming that despite linguistic, cultural, and individual differences, emotions can be correctly recognized in the facial expressions and gestures of all people [53]. However, not all computer systems can take advantage of technical solutions to interpret facial expressions and gestures. Such a situation occurs in the currently very popular Contact Center (CC) systems, where the customer’s interaction with the agent often takes place exclusively via voice or text channels.

If we want to interpret the behavior of people interacting using CC systems and, on the other hand, improve the effectiveness of voice and text communication, it seems sensible to use emotion recognition methods based on audio [7,8,9,54] and/or text [31,55] analysis. Typically, the capabilities of artificial intelligence (AI) methods are used in this regard, particularly in the areas of machine learning (ML) and natural language processing (NLP) methods [56]. It is worth noting that the known computer systems make it possible to effectively identify only selected types of affective states based on the sound signal or text data. In most solutions, these are generally referred to as positive, neutral, and negative states. On the other hand, solutions that recognize specific types of emotions are also known, for example, disgust, openness, happiness, frustration, sadness, fear, surprise, and anger [57,58,59]. In addition, a common problem is interpreting them appropriately, taking into account the context of the conversation. The virtual assistant, which is more and more eagerly used in CC systems, fulfills its intended function as long as it can recognize the intentions of contacting customers. However, the interpretation of customers’ true intentions can be hindered by the emotions they display during the conversation. Therefore, from the point of view of CC industry needs, an important issue is to develop functionality for bots to distinguish the affective states of callers on hotlines. According to the authors of the article, the first stage of work in this area is to develop an *Emotion Classification for Machine Detection of Affect-Tinged Conversational Contents*.

Currently, many professional software tools are known to support the construction of virtual assistants [60,61]. Examples include Google DialogFlow, Amazon Lex, IBM Watson, Wit.ai, Microsoft Azure Bot, or RASA. Except for Google Dialogflow, none of the tools mentioned have the functionality to recognize customers’ affective states. Google Dialogflow analyzes the emotionality of users’ statements to some extent. However, the offered solution determines affective states only based on text data, without considering acoustic parameters of the sound signal, containing a lot of important information about the emotional state of the interlocutor. Furthermore, this solution only identifies positive or negative affect, without recognizing specific types of emotions [62]. For CC systems, limiting procedures to only identifying positive and negative affect significantly limits the ability to improve customer service. In contrast, accurately identifying customer emotion types can contribute to a significant reduction in service costs, as measured by selected KPIs (key performance indicators) [63]. KPIs that can be improved as a result of implementing a new emotion recognition procedure include, for example, improved C-SAT (customer satisfaction) or FCR (first call resolution), which measure the number of cases resolved during the customer’s first contact with the CC.

Due to the nature of the services provided by CC systems, the implementation of effective IT solutions for the recognition of affective states is much needed. Nevertheless, it seems important to identify the types of emotions that bring the most tangible benefits in the indicated application area. To the best of this article’s authors’ knowledge, existing research does not address these issues, which is the motivation for the following effort: (1) development of the *Emotion Classification for Machine Detection of Affect-Tinged Conversational Contents* in Contact Center Systems and (2) verification of its usefulness based on real material from a large CC system (recorded phone calls and chat conversations). The contribution to the body of knowledge of this paper is as follows:

▪ Development of the *Emotion Classification for Machine Detection of Affect-Tinged Conversational Contents* directly dedicated to the CC industry;
▪ Verification of the developed *Classification* based on the developed algorithms for machine detection of customers’ affective states in CC voice and text channels.

Given the assumptions discussed above, the study pursued the following objectives. First, based on material from CC voice and text channels, an attempt was made to develop the *Emotion Classification for Machine Detection of Affect-Tinged Conversational Contents*. Second, an attempt was made to develop efficient procedures for machine detection of customer emotions in CC voice and text channels. Third, an attempt was made to verify the usefulness of the *Classification* in machine detection of customer emotions in CC voice and text channels. Finally, the developed *Classification* was recommended along with the artificial intelligence algorithms that gave the best results.

## 2. Method

The proposed *Classification* was developed based on samples of real conversations realized in the voice and text channels of the CC system. In the first step, the empirical material was evaluated by three specialists. Depending on the communication channel, they were: a psychologist dealing with the topic of emotions, a linguist, and specialists in computer-aided emotion detection. These individuals developed a working classification of emotions found in the speech samples analyzed, based on a frequency analysis of CC customers’ affective states. In the next step, the usefulness of the developed *Classification* was verified taking into account the technological capabilities of emotion recognition in computer systems. For this purpose, the developed emotion recognition method, dedicated directly to the CC industry, was used, based on artificial intelligence solutions, including machine learning procedures and natural language processing. As already mentioned, automatically determining emotions for the purposes of CC systems is a relatively difficult task. It should be emphasized that audio signals typically found in CC systems are of much lower quality than studio or radio recordings, and they are extremely varied and often distorted, which makes it very difficult for artificial intelligence algorithms to evaluate them correctly. Therefore, algorithms were used to normalize the volume levels of individual calls for the purpose of correcting and normalizing selected parameters of the examined signals. All conversations examined in this study were conducted in Polish.

### 2.1. Samples

Analyses were conducted using two independent samples, including data from one of the largest CC systems in Poland, which has been operating for several dozen years and handles a variety of commercial customers, including telecom operators, insurance companies, postal carriers, and taxis. *Sample 1* included two independent databases. The first base consisted of customer conversations with agents in the voice channel (phone calls), while the second base included data from the text channel (chat conversations). The databases were completed by including conversationally drawn phone calls and chats recorded on selected days of the week between July and December 2020. It was decided that the research samples would be randomly drawn from the six-month period of operation of the CC system to ensure that the conversations were adequately represented and that the data collected could be reliably evaluated. For the purpose of analysis, three topics of conversation were selected, defined as (a) invoices and payments, (b) technical information, and (c) contracts and annexes. In total, the first base contained 302 phone calls, while the second base contained 345 chats. The duration of voice calls ranged from 3 to 20 min. The total length of the recordings was 22 h 59 min and 11 s. Therefore, the average length of the conversations was 4 min and 36 s. In the second base, chat conversations were collected, where each drawn conversation contained a minimum of 300 characters. The total number of customer and agent contributions in the text channels was 7515; hence, the average number of contributions per chat was 21. Based on the material extracted from the voice and text channels of *Sample 1*, the *Emotion Classification for Machine Detection of Affect-Tinged Conversational Contents* in CCs was developed. The data collected in *Sample 1* also served as teaching and testing data for the developed machine learning algorithms.

In addition, *Sample 2* was prepared to validate the developed algorithms and to verify the *Emotion Classification* proposed in the paper. Similarly to *Sample 1*, it contained two independent databases labeled as base three and base four. Base three contained 100 conversations conducted in a natural context, while base four contained 100 chat conversations. All verification data were randomly selected from the same CC system between January and March 2021 and were related to the same interview topics. The total length of the recordings was 7 h 25 min and 15 s. Therefore, the average length of the conversations was 4 min and 27 s. Conversations ranging in duration from 3 min to a maximum of 20 min were selected. The total number of customer contributions in text channels was 3718, averaging 37 contributions per chat. Each selected chat contained a minimum of 300 characters.

All conversations in both *Samples* were anonymized (data sensitive from the point of view of the GDPR—General Data Protection Regulation—were removed) before being submitted for research purposes. Based on the data from *Sample 2* and the developed algorithms, the emotional states occurring during the conversation between the customer and the agent were determined. The obtained results were compared with the ratings proposed by three independent judges in the next step. Based on the findings, the usefulness of the *Emotion Classification* proposed in the article was verified.

### 2.2. Testing Procedure

#### 2.2.1. Emotion Classification—Voice and Text Channel

Analyses in sound channels were performed in two ways. First, the *Emotion Classification* was developed. For this purpose, three judges (two psychologists dealing with the topic of emotions and an engineer designing algorithms for detecting emotions in voice channels) independently extracted affectively colored fragments from the phone calls (*Sample 1*). Emotions expressed by CC customers in telephone conversations were identified based on established acoustic indicators. For anger and derivatives (e.g., rage, sarcasm, irony, etc.), judges considered the following acoustic parameters [64,65]: (1) an increase in the mean *f*_0_ (*f*_0_—fundamental frequency), (2) its variance and (3) its range, (4) an increase in the intensity of the speech, and (5) an increase in the high-frequency energy and (6) intensity. For fear and derivatives (e.g., panic, apprehension, anxiety, etc.), they considered the following [66,67]: (1) an increase in mean and (2) baseline *f*_0_ and (3) its range, (4) an increase in intensity and (5) high-frequency energy, and (6) an increase in articulation rate. For sadness and derivatives (e.g., melancholy, grief, bitterness, etc.), the following were considered [66,68]: (1) a decrease in mean and (2) baseline *f*_0_ and (3) its range, (4) a decrease in intensity, (5) low high-frequency energy level, and (6) a decrease in articulation rate. For happiness and derivatives (e.g., contentment, happiness, relaxation, etc.), the following were considered [69,70]: (1) an increase in mean and (2) baseline *f_0_*, (3) its range and (4) variance, and (5) an increase in intensity and (6) rate of speech.

The judge identified an emotion if the fragment contained at least three characteristic indicators of that emotion. Then, during the next listening session, the judge paid attention to the content of the customer’s statements. If the semantic content was consistent with the previous detection, then the fragment went into the pool. Otherwise, it was omitted. Judges’ detections were then compared—when an excerpt in terms of location in the file and duration of +/− 2 s was rated in agreement by the judges, affective coloring ratings were analyzed in the next step. Finally, fragments that were consistent in terms of their formal characteristics and content assessments of affective coloring were entered into the pool. A total of 400 utterance fragments were left in *Sample 1* after selection to identify the specific type of emotion present. On the other hand, if the analyzed conversation did not contain fragments tinged with affect (i.e., it was devoid of at least three acoustic parameters and there was no emotion-specific content, e.g., “how happy I am to have solved the problem” for happiness, etc., or “I am unhappy,”, etc., for anger), the conversation was identified as neutral. Finally, 129 whole files (conversations) were entered into the pool where only the neutral state was identified. In order to carry out the learning and testing processes using artificial intelligence algorithms, the data fragments selected by the judges were divided into fragments of a few seconds, which ultimately allowed the construction of a set consisting of 2935 elements. The prepared collection included all identified affective states. Its detailed analysis is presented in Table 2.

The analysis of conversations in text channels was also performed in two ways. Three judges (a linguist and a psychologist dealing with the topic of emotions, and an engineer designing algorithms for detecting emotions in text channels) independently extracted fragments tinged with affect from the chats (*Sample 1*). Emotions expressed in chats by CC customers were identified based on established semantic, syntactic, vocabulary, orthographic, and iconic indicators. For anger and derivatives (e.g., rage, sarcasm, irony, etc.), the judges considered the following parameters: (1) direct naming of the accompanying emotion, e.g., I am furious, angry; (2) occurrence of emotionally charged words: insults, vulgarisms, coarsening; (3) type of sentence: indicative, imperative, exclamatory; (4) use of diacritics (period, exclamation mark); (5) font type and weight; (6) type of emoticon: >:(, >:-(, :P, :-P, :/, :-/ [71,72]. For fear and derivatives (e.g., panic, apprehension, anxiety, etc.) they considered the following: (1) indication of the accompanying emotion; (2) use of fear-indicating lexemes: excuse me, what now, exclamation marks(oh shoot, ah), onomatopoeias; (3) type of sentence: indicative, question, exclamation; (4) diacritical marks: exclamation marks, question marks, colons, pauses; (5) font type and weight: neutral, unhighlighted; (6) type of emoticon: :O, :-O, :o, :-o [73,74,75]. For sadness and derivatives (e.g., melancholy, grief, bitterness, etc.), the following were considered: (1) indication of the accompanying emotion; (2) use of lexemes indicating fear: unfortunately, it doesn’t work, I don’t know, whoops; (3) type of sentence: disjunctive coordinate, negative; (4) diacritical marks: question marks, ellipsis; (5) font type and weight: neutral, undistinguished; (6) type of emoticon: :(, :-(, :((, :-((, :’(, :’−([74]. For happiness and derivatives (e.g., contentment, happiness, relaxation, etc.), the following were considered: (1) indication of the accompanying emotion; (2) use of lexemes indicating happiness: great, kisses, thank you, superb; (3) type of sentence: indicative, exclamatory; (4) diacritical marks: exclamation point, period; (5) font type and weight: neutral, highlighted; (6) type of emoticon: :), :-), :)), :-)), :D, xD, XD [76].

As with the analysis of the audio files, the judge determined the emotion if the excerpt contained at least three indicators characteristic of it. The judges’ detections were then compared—when a fragment was rated in agreement by the judges, then it went into the pool. Finally, 1480 utterance fragments identifying each emotion type and 2269 fragments identifying the neutral state were entered into the pool. A detailed analysis of the prepared base is presented in Table 3.

#### 2.2.2. Machine Emotion Detection

The developed emotion recognition algorithms were based on artificial intelligence methods in machine learning and natural language processing. These methods include advanced models that are used to solve regression as well as classification problems, where there are usually many input as well as output variables.

In terms of audio data, the following speech signal descriptors were tested for optimal use during the construction of the method: frequency and spectral characteristics, *mel*-frequency cepstral coefficients, and Linear Predictive Coding (LPC) coefficients. As a result of the analyses, a final group of audio signal parameters is selected that produces the best results in the emotion determination process for the test audio dataset *Sample 1*. The following parameters were selected for the speech signal: *f*_0_ (fundamental frequency), MFCC (Mel Frequency Cepstral Coefficients), jitter and shimmer. It was determined that the first 13 MFCC coefficients describing the frequency parameters of speech would be used for emotion recognition because they contain most of the information regarding the emotion to be recognized [77]. The fundamental frequency *f*_0_, on the other hand, contains information about the pitch of the voice, and therefore allows us to take, e.g., gender into account, without the need for additional determination. These features are extended to include voice frequency changes during the speech, i.e., the jitter parameter, and amplitude changes described by the shimmer parameter. In the initial phase of the machine emotion detection process, the following classifiers were used: Support Vector Machine (SVM), k-Nearest Neighbor (kNN), Convolutional Neural Networks (CNN) and Linear Discriminant Analysis (LDA). Finally, the LDA classifier was discarded due to unsatisfactory results. 

The choice of parameters for machine learning algorithms is highly dependent on the data used in the problem being solved. Therefore, it is important to analyze the effect of individual classifier parameters on the performance and efficiency of the developed models [78,79]. Thus, for the algorithms tested in both communication channels, extensive research related to the optimization of their parameters was performed. For the voice channel, the best results in the *Sample 2* based verification process were obtained for CNN. The CNN network structure consists of 13 layers. These are, respectively: a spline layer (which creates an input data tensor of size 19 consisting of 256 output filters in the spline), a linear Relu activation function layer, a spline layer consisting of 128 output filters in the spline, another linear Relu activation function layer, a dropout layer (sets the input units in each learning step with a step of 0.1 to avoid over-fitting), a MaxPooling1D layer for temporal data of size 8, another spline layer consisting of 128 output filters in the spline, another layer of linear Relu activation function, another spline layer consisting of 128 output filters in the spline, another linear Relu activation function layer, a Flatten layer (flattening the input data to one dimension), a deep layer with 5 nodes, and a Softmax activation function layer and an RMSProp-type optimizer. The following value ranges were analyzed for the proposed approach: input size is chosen from {13, 18, 19, 24}—depending on parameter configuration for {MFFC—13}, {MFCC + Jitter—18}, {MFCC + Shimmer—19} and {MFCC + Jitter + Shimmer—24}, activation function is selected from {‘relu’, ‘sigmoid’}, batch size is chosen from 8 to 128 (best value 16), learning rate is selected from {0.0001, 0.0005, 0.001, 0.01} (best value 0.001) and epochs number is selected from 50 to 2000. In order to avoid over-fitting the network, the effect of the learning parameters on the convergence of the algorithm and the error function values obtained for both the learning/testing and validation data were also evaluated in successive epochs. The implemented CNN obtains the best results for 1000 learning epochs. The SVM classifier used a Radial Basis Function (RBF) kernel and an L2 regularization with a weight of 1.0. For the kNN classifier, the best results were obtained with the number of neighbors equal to 5, the Euclidean distance and with uniform weights.

In contrast, text conversation emotion classification algorithms were based on property vectors. This technique allowed semantic analysis of words given the context of their occurrence. The property vector model was trained from scratch on data extracted directly from transcribed chat conversations to account for content that has emotional valence. The ability to use actual CC conversations in the verification process was a great advantage, as publicly available word sets are mostly based on ordinary texts, which by their nature do not contain much emotional content. With the property vectors representing the learning set, machine learning models were then trained to recognize specific emotion types. For this purpose, the following models were considered: an Artificial Neural Network containing bidirectional recurrent layers based on LSTM cells (ANN), a Random Forest Classifier (RFC), a Support Vector Machine (SVM) classifier, a Decision Tree (DT) classifier, an Artificial Bee Colony (ABC) classifier, and a k-Nearest Neighbor (kNN) classifier and Quadratic Discriminant Analysis Classifier (DAC). Finally, two of the aforementioned solutions, namely ABC and DAC classifiers were discarded during the ongoing investigations due to unsatisfactory results. In the case of the classifier using artificial neural networks, training and classification using complete texts fed to the input of the network were possible. However, due to the nature of the other classifiers, additional preprocessing of the input data was necessary. For this purpose, the Term Count (TC) vectorization technique based on counting occurrences of keywords with emotional connotations in the text was used. To improve the performance of this technique, keywords that occur in all texts regardless of the emotion present were also included. Inverse Document Frequency (IDF) techniques were used for this purpose.

The architecture parameters of the various classifiers used in the process of emotion recognition from data in text form that produced the best results were as follows. For ANN, the network structure consists of 9 consecutive layers. They are, successively: a word embedding layer, a BLSTM layer with 50 nodes, a dropout layer with step 0.1, a deep layer with 300 nodes, a linear Relu activation function layer, a dropout layer with 0.1 step, a deep layer with 120 nodes, a linear Relu activation function layer, a dropout layer with 0.1 step, a deep layer with 5 nodes, a Softmax layer, and an Adam-type optimizer (a stochastic gradient descent optimizer using adaptive first- and second-order moment estimation) with a learning rate of 0.001 and a categorical loss function kross entropy. For the kNN classifier, the best results were obtained with the number of neighbors equal to 5 and the Euclidean distance with uniform weights for all samples. For DT, Gini coefficient was used, no maximum depth criterion, splits were performed when a leaf contained more than two samples. In contrast, for the RFC classifier, 100 random trees were created, Gini coefficient was used, no maximum depth criterion, splits were performed when a leaf contained more than two samples. For the text channel, the best results in the *Sample2* based verification process were obtained for the SVM classifier. For SVM, kernel is selected from {‘linear’, ‘poly’, ‘rbf’, ‘sigmoid’}. Gamma is chosen from {‘auto’, ‘scale’}. Shrinking and probability are selected from {true, false}. Catch size is chosen from {100, 200, 500, 1000}, decision function shape from {‘ovo’, ‘ovr’} and tolerance is chosen from {0.00001, 0.0001, 0.001, 0.01, 0.1, 0.2, 0.25, 0.3, 0.4, 0.5, 0.6, 0.7, 0.8, 0.9, 1, 1.1}. In our solution, the following values are chosen: kernel = ‘rbf’, gamma = ‘scale’, shrinking = ‘true’, probability = ‘false’, cache size = 200 MB, decision function shape = ‘ovr’, tolerance = ‘0.001′, and there was no limit for maximum iteration.

Using the data covering *Sample 1*, the learning and testing processes of the selected classifiers were conducted. For this purpose, the data of *Sample 1* (both the sound recording database and the chat database) was divided into two sets, the teaching data set and the testing data set. The teaching data comprised 80% (2348 recording excerpts and 2999 text excerpts) and the testing data comprised 20% (587 recording excerpts and 750 text excerpts) of the samples from actual conversations conducted in the CC system selected for testing. Balancing techniques were used to divide the data into learning and testing sets. The problem of unbalanced data in classification processes arises when the sample prepared for research is characterized by large discrepancies in abundance between classes. This situation occurs in the actual CC conversations studied in the paper. In the conducted study, the Synthetic Minority Over-Sampling Technique [80] was used. This allowed us to optimize the parameters of the emotion detection method used, which in turn had a positive impact on the final classification results. Five-fold cross-validation was performed to evaluate the built models.

Ultimately, for both communication channels (voice and text), the emotions determined by the Judges for *Sample 1* were compared to the emotions recognized automatically by the classifiers used in the system. To determine the effectiveness of the classification models, the following metrics were determined: *ACCURACY*, *PRECISION*, and *F1-Score*. *ACCURACY* is the most common metric used to assess the quality of classification; however, since in the case under consideration the element counts in each class are not balanced, the results obtained had to be additionally verified using weighted metrics. Thus, the weighted metrics *PRECISION* and *F1-Score* were included in the efficacy assessment. The *RECALL* metric was not included because its weighted values are the same as the *ACCURACY* metric. The results of this study are presented in Section 3.2 in Table 4 and Table 5.

#### 2.2.3. Assessing the Usefulness of Classification in Machine Detection of Emotions

In the process of final verification of the proposed *Classification* usefulness in machine detection of emotions, the prepared *Sample 2* was used. These data were not involved in the learning and testing process—it was only for the purpose of verifying the *Classification*. The developed automatic emotion detection method indicated the emotional states of both interlocutors (agent and customer) during their real conversations in voice and text channels. The emotional states recognized by the system for *Sample 2* data were then independently verified by individual judges according to the assumptions described in Section 2.2.1. This allowed us to determine appropriate metrics for each Classification to verify detection efficacy, as presented by the results in Section 3.2, in Table 6.

## 3. Results

### 3.1. Emotion Classification for Machine Detection of the Affective Coloring of Conversational Content in Voice and Text Channels of CC Systems

To develop the Classification, the judges independently conducted a frequency analysis of the emotions that were identified in the pre-defined excerpts from the phone statements and chat conversations. As a result of the final discussion among the judges, a single score was established for the extracted parts of utterances. Finally, the audio and text files (also used in the learning and testing of the classifiers) were added to the pool of files for which there was 100% agreement in the judges’ evaluation. Due to the adopted evaluation procedure, the concordance coefficients of the judges’ evaluations for the audio and text files (e.g., alpha-Krippendorf) were equal to 1. To reduce the number of recognized emotions, they were assigned to broader classes, the so-called basic emotions [16,81] and their corresponding derivatives, the so-called emotion families [82]. The size of each class of affective states in the voice channels were as follows: anger = 937, fear = 166, happiness = 196, sadness = 145, neutral = 1491. In contrast, for the text utterances, the following total values were selected: anger = 312, fear = 102, happiness = 761, sadness = 305, neutral = 2269. As can be seen, the judges also succeeded in identifying affectively neutral conversations, according to the plan providing a baseline for the machine analyses conducted in the next step. Table 2 summarizes the results obtained by compiling the descriptive statistics of the basic emotion classes and neutrality, the corresponding family of related emotions identified in Sample 1 of the database containing data from the voice channel along with real examples of utterances illustrating a specific affective state.

Analyzing conversations for the length of recordings in which the customer’s affective states were identified, it was found that neutral state statements were the longest in the sample (63.57% of Sample 1 duration). Such a situation is completely natural and expected. This was followed by the detection of anger (5.74%), fear (2.68%), sadness (0.53%), and happiness (0.24%). In the analyzed conversations, affective states were recognized in recordings with a total duration of 16 h 43 min 25 s, which represents 72.75% of the total duration of all collected conversations (22 h 59 min 11 s). In the remaining recordings of 6h 7 min 53 s (27.75% of total time), affective states were not clearly defined by the judges, resulting in their exclusion from further study. This is primarily due to the specifics of the CC conversations (natural conversations, often with additional distractions). Table 3 summarizes the findings on frequency analysis of the emotional coloring of the CC consumers’ speech fragments based on the text channels of Sample 1.

In conversations originating from the text channel, as expected, neutrality was the most common affective state (30.20%). This was followed the detection of happiness (10.12%), anger (4.15%), sadness (4.05%), and fear (1.36%). Customers were more likely to show happiness in text channel calls than in voice channel calls because they were more likely to seek contact for issues that an agent can resolve right away (e.g., top-up, check account balance). At the same time, 3766 (50.12%) utterances were not uniquely identified by the judges, the reason being too few characters, inarticulate phrases occurring, or texts resulting from the hashing of sensitive information. These utterances were not entered into the pool.

### 3.2. Findings on Machine Detection of Emotions in CC Voice and Text Channels

The evaluation of models prepared for machine detection of affective states in CC voice and text channels was performed using Sample 1 data. Table 4 shows the results of automatic detection of affective states in the voice channels, while Table 5 shows the results in the text channels. These data present the average results obtained through a five-fold cross-validation process, confirming the stability of the models tested.

Analyzing the data presented in Table 4 and Table 5, it can be seen that the average cross-validation values for the voice channel range from 60% to over 80% depending on the classifier. Thus, this confirms the validity of the developed models. For the text conversation analysis, the classification results oscillate between about 50% and 66%. The better results obtained in the classification process of the audio data were expected due to its nature of allowing the use of a wide range of descriptors. In contrast, the data from Sample 2 were used to verify the prepared solution, for which the classification results are presented in Table 6.

For the verification sample, the best results were obtained using the following classifiers: (1) CNN—for the voice channel; (2) SVM—for the text channel. At the same time, it can be seen that other classifiers yield results that are relatively not much weaker, so they are also worth considering in potential further work involving the development of the research topic described.

## 4. Discussion

In this paper, based on the analysis of conversation fragments from CCs, *The Emotion Classification for Machine Detection of Affect-Tinged Conversational Contents* in voice and text channels of CC systems was developed, which distinguishes five basic affective states—anger, fear, happiness, sadness and neutral. From the findings presented in Section 3, it can be seen that the effectiveness of machine classification of affective states for the text channel is significantly higher than 50%, and for the voice channel it is higher than 60%. Pawlik et al. [61] demonstrated that the emotion detection method used by the authors provides a level of performance in recognizing affective states that significantly improves the performance of virtual assistants. This also confirms the usefulness of the *Emotion Classification for Machine Detection of Affect-Tinged Conversational Contents* proposed in the paper in applications for CC companies.

**Figure 1:**
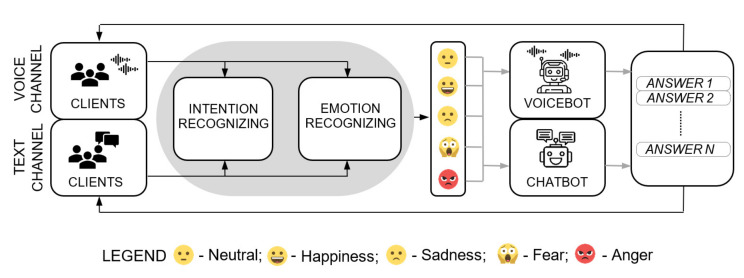
How virtual assistants function taking into account the recognized emotions.

As shown, machine-based emotion detection performed by artificial intelligence algorithms can be effective in CCs. Knowledge of the emotions occurring during a customer-agent conversation is especially important when a chatbot or voicebot plays the role of an agent. Virtual assistants find increasingly frequent use in CC systems, replacing individuals in solving increasingly complex problems [83]. As indicated earlier, for the effectiveness of a virtual assistant to be high, it must be able to correctly identify the intentions of the customer talking to it. Intent detection methods are used for this purpose. However, for emotionally charged conversations, actual intentions can be very difficult for machines to discern. In human-to-human conversations, interpreting words or phrases in conjunction with a particular type of emotion often completely changes the direction of the conversation, which is challenging with virtual assistants. This is related to the understanding of the affective messages conveyed by the customer verbally, but also emotionally. The same phrases uttered with a particular emotion tend to have a different semantic meaning than the same phrases without an emotional background [84]. Therefore, the task of virtual assistants should be to conduct conversations in a way which takes into account previously identified emotions, if any. Then, the bot will be able to treat a phrase with a negative emotion differently than the same phrase with positive emotion, or a neutral phrase. Such a process can be accomplished precisely by integrating emotion detection methods with intention detection methods. Considering the role of emotions, which often form the backbone of intent in individual conversations, will significantly increase the level of understanding of the customer’s speech by the machine, resulting in an increase in the number of cases resolved. Figure 1, in the form of a flowchart, shows how virtual assistants function in the voice and text channel taking into account the recognized emotions.

Knowledge of the emotions that occur during a customer-agent conversation is also very important when creating algorithms designed to match callers in the most optimal manner. In CC systems so far, callers are usually matched only from the point of view of their skills. Knowledge about emotions, on the other hand, will allow caller-matching algorithms to be built taking into account callers’ personality traits. Such algorithms should use specific behavioral profiles of communicators. To create behavioral models, however, it is necessary to be able to automatically recognize emotional states. Intensive research is currently being conducted by the authors in this area. In addition, knowledge of an agent’s emotions can be useful to supervisors of CC campaigns. The supervisor will receive a message when characteristics indicating the appearance of negative emotions are detected, allowing for a quick and appropriate response. Currently, performance evaluation of agents is usually conducted by reviewing randomly selected conversations. Functionality that analyzes emotional states can greatly improve the effectiveness of Supervisors’ work. Another element that should be emphasized is the ability to use behavioral profiles of agents in predictive staffing scheduling.

## 5. Limitations

The limitations of the study described here are specific to research on the detection of emotions expressed via voice and text. The following sections present the possible limitations of the conducted study, identified by the authors, which may also be potential areas for further research work. The following limitations were identified:

▪ For voice conversations conducted in natural context, noise interference from the environment can affect the correctness of emotion detection by the judges, as well as the performance of the developed system. Due to the typically poor quality of recordings and lack of standardization, detection based on listening to natural utterances (including those from CC systems) is far more difficult than detection based on voice samples prepared by trained actors for experiments [16,70]. The recorded voice strength in natural conversations, as well as a number of other parameters, is not necessarily indicative of, for example, a person’s arousal, but of the quality of the telephone call, the quality of the equipment used for the call, the distance of the handset from the mouth, and many other important interfering variables. Moreover, actors’ utterances are usually exaggerated, and experimental samples are abstracted from the natural context of the utterances, i.e., their place, purpose, etc., i.e., deprived of information critical for the direction of interpretation of the expressed emotions [70,85];
▪ Similar problems can also be encountered in the case of written text samples, e.g., when a person, due to time pressure, simplifies the message and makes syntactic and grammatical errors, which can then significantly affect the detection of emotions by the judges as well as the information technology system;
▪ The accuracy of emotion detection by judges and then the system may be higher for intentionally prepared materials, where emotions are induced under standardized conditions using pre-prepared affective stimuli, e.g., excerpts from horror films, comedy, etc., compared to natural conversations recorded in CCs as an example [82]. It follows that the results of studies that use intentionally prepared material have higher relevance but lower ecological accuracy [86]. In contrast, results from studies based on conversations conducted in natural settings have relatively lower reliability but higher ecological accuracy and, therefore, generalizability to other natural settings and contexts [87];
▪ The cultural context of the study may be an important limitation. It cannot be ruled out that certain types of emotions may be more or less easily expressed in speech and writing, and consequently recognized, within specific groups of languages, e.g., Slavic vs. Germanic, Romance, Semitic, etc. Furthermore, speakers of different languages may have different sensitivities to specific groups of emotions expressed in speech or writing [88,89]. The problem discussed therefore concerns the representativeness of the results obtained. Perhaps, based on conversations conducted in a language other than Polish, different results would be obtained, contrasting with those presented. This issue is worth addressing in future research.
▪ It is worth noting that the field of psychology has also developed a concept that makes the strength of emotional reactions dependent on the language used at a given moment. For example, Zajonc [90] noted that humans are often unaware of the role of emotions in decision making—especially under conditions of uncertainty. This process does not occur as strongly in a foreign language as in the native language. A phenomenon called the foreign-language effect has been defined as a result of research that has shown weaker emotional responses when content is processed in a language the user is less fluent in rather than the native language [91]. Despite understanding its meaning, a language user always perceives more emotionally a message formulated in the native language than in a foreign language, which is supposed to promote the rationalization of thinking in a foreign language. The reason for the foreign language effect is most likely due to the lower level of emotional reactions felt when thinking in a foreign language [92].
▪ Interview samples were obtained within the three interview topics mentioned earlier (invoices and payments; technical information; contracts and annexes). As such, it is difficult to assess how representative the results derived from them are of the entire CC context and, more broadly, how typical the collected sample of CC conversations is of contexts other than the CC system. This issue can be considered in the context of further research on the topic discussed in the paper. To be able to conduct this research, it may be necessary to optimize selected components of the authors’ emotion recognition method used. Among other things, for conversations in the text channel, the integrated dictionary of emotional expressions may need to be supplemented to some extent. This dictionary was implemented as a dedicated component of an emotion recognition method designed for both text channels and audio channels. In audio channels, the ability to use the dictionary is provided by automatically generated conversation transcripts. The ability to additionally include transcripts of conducted audio conversations increases the effectiveness of classification. Nevertheless, the transcription method used dedicated to the CC industry [60] was optimized for the above three topics. Therefore, another component that may require potential optimization is the integrated transcription method. The method would primarily need to optimize the algorithms operating on the post-processing side. In addition, appropriate learning processes can be implemented to make the solution even more versatile. The task of these solutions should be to continuously and dynamically adjust the reference models used. Intensive work is currently being carried out in this area, which is focused on the following possibilities: (a) the addition of learning processes to the existing model and (b) the preparation of a new model based on additional learning data, for which the classification results are considered taking into account the results obtained for the original model. Consideration of new learning data, particularly the amount of data, may further impact the need to optimize the data balancing techniques used. The potential activities listed above illustrate the additional work that may need to be conducted when implementing a solution for a completely different industry.
▪ Not all emotion types known in psychology are identifiable to a satisfactory level of efficiency using intelligent computer systems. This limitation significantly narrows the possibilities in the development of the *Emotion Classification for Machine Detection of Affect-Tinged Conversational Contents* serving the needs of CC systems.

## 6. Conclusions

The literature analysis presented in this paper revealed that studies on emotion detection in the context of CC systems, despite claims of validity, are sparse and selective. Additionally, they are characterized by methodological limitations due to language specificity and mainly focus on identifying potential benefits related to improving customer service quality, without pointing to specific solutions. Considering the obtained results and the limitations described in Section 5, the authors recommend that the CC industry use the *Emotion Classification for Machine Detection of Affect-Tinged Conversational Contents*, which includes the following emotions: anger, fear, happiness, sadness, and neutral. As the research shows, they are important for practical applications and are often found in many CC conversations.

Most people who contact CCs hope for a successful resolution of issues that stir up different emotions in them. When a problem is resolved positively, there is often happiness in the customers. In other situations, we see, for example, fear resulting from, e.g., a damaged phone, or sadness resulting from, e.g., a lack of Internet connection. Customers also contact CC out of anger, which is often caused by lost hope for a quick resolution to a problem, such as a customer complaint about a product or service. In all cases, accurately identifying the customer’s emotions and then effectively using knowledge about them seems to be a key factor for the course and final results of the interaction. The solutions proposed in this paper for machine detection of the affective coloring of conversational content in voice and text channels of CC systems serve these purposes.

Further work on the development of the proposed topic includes the continuation of research related to the development and implementation of the prepared voicebots and chatbots, which will ultimately be used in various thematic CC campaigns. The solutions will use machine-detected emotions recommended in the paper. It is also anticipated that the research topic proposed in this paper can be used in CC systems of the future, where various video and transmission technologies will become significantly more important in customer service [93,94]. These technologies could also support emotion detection processes or even extend the scope of the *Classification* proposed in this paper. In addition, it is possible to use the discussed topic to build behavioral profiles of both customers and CC agents.

## Figures and Tables

**Table 1:** Selected systems for automatic recognition of affective states.

System	Affective State	Parameters	Technical Solutions	References
An analysis of the emotions of people using intelligent tutoring systems (ITS)	Anger, neutrality, disgust, sadness, fear, happiness, surprise	Facial expressions, skin conductivity	Sensor electrodermal activity (EDA), face reader, recording camera, neural network	[46]
Dynamic facial expression recognition sequence	Neutrality, disgust, sadness, fear, happiness, surprise, anger	Facial expressions	Convolutional Neural Network (CNN)	[47]
Detection of emotions based on computer analysis of body posture	Lividness, boredom, disgust, happiness, interest	Body posture	Algorithm on C++, a sensor that analyzes body posture	[21]
Detection of emotions based on gestures and body movements	Happiness, sadness, surprise, fear, anger, disgust and neutral state	Gestures and body movements	A sensor that analyzes posture, Convolutional Neural Network (CNN)	[22]
Moodies for voice-based emotion detection	Disgust, happiness, anger, fear, tenderness and sadness	Sound	An app that detects the emotion in people’s voice	[7]
Techniques for recognizing emotions from voice	Anger, happiness, sadness and neutral state	Sound	Deep neural networks, hybrid CNN and SVM model	[8]
A prototype system for detecting emotions in a text based on social media posts	Anger, anticipation, disgust, fear, joy, sadness, surprise, trust	Text	Long Short Term Memory (LSTM) networks	[15]
A model for emotion recognition based on ECG signal analysis	Happiness, sadness, pleasure, anger	ECG	Spiker-Shield Heart and Brain sensor, Extra Tree Classification, ADA Boost Classification with SVM, Python Scikit API	[25]
Development of an emotion recognition system based on physiological reactions of the organism	Sadness, fear and pleasure	ECG ^1^, GSR ^2^, BVP ^3^, pulse, respiration	A system with five physiological signal sensors (ML870 PowerLab 8/30 sensor), Support vector regression (SVR)	[27]
Emotion Recognition Using Heart Rate Data from a Smart Bracelet	Happiness, sadness and neutral	Pulse	A smart bracelet (Algoband F8), k-Nearest Neighbor (kNN), Random Forests (RF), Decision Tree (DT), Gradient Boosting Decision Tree (GBDT), Adaptive Boosting	[29]

^1^ ECG, electrocardiogram; ^2^ GSR, galvanic skin responses; ^3^ BVP, blood volume pulse.

**Table 2:** Frequency analysis of the emotional coloring of utterance fragments in *Sample 1* (voice channel).

Affective State	Descriptive Statistics		Family of Related Emotions
	N(%) ^1^	L(%) ^2^	
**Anger**	937 (32)	1 h 19 min 7 s (5.74)	irritation, impatience, negative surprise, disappointment, bitterness, anger, irony, sarcasm, rage
*Examples: “This agreement is ridiculous, it’s not insurance at all;” “Wait, wait, are you kidding me?! This is complete nonsense;” “Why do you cheat people like this?! I’ve legitimately been duped.”*			
**Fear**	166 (5)	36 min 58 s (2.68)	uncertainty, fear, worry, confusion, anxiety, panic
*Examples: “It’s taking an awfully long time, could you please hurry up?;” “This sort of thing shouldn’t happen because the GDPR is in force now;” * *“I’ve tried to explain but there seem to be problems, I’m having a problem.”*			
**Happiness**	196 (7)	3 min 16 s (0.24)	interest, satisfaction, positive surprise, excitement, gratitude, hope, happiness, amusement
*Examples: “I am very happy, this is great, thank you very much;” “Thank you kindly, you helped me a lot, have a nice day;” “Okay, let’s do it this way, I am very happy, everything has been explained.”*			
**Neutral**	1491 (51)	14 h 36 min 46 s (63.57)	not applicable
*Examples: “Okay, thank you for the information;” “Yes, that’s right, I understand everything;” “I would like to know something about my complaint. Can we please check it out? Thank you.”*			
**Sadness**	145 (5)	7 min 18 s (0.53)	resignation, bitterness, helplessness, regret, melancholy
*Examples: “Oh well, that’s too bad, if it can’t be handled otherwise;” “It’s a bit unfair (…) This doesn’t sit square with me;” “And now what? I don’t want that phone, I don’t understand, this is impossible”*			

^1^ N(%), total number of fragments and their percentage in the sample; ^2^ L(%), total duration of fragments and their percentage in the sample.

**Table 3:** Frequency analysis of the emotional coloring of utterance fragments in Sample 1 (text channel).

Affective State	Descriptive Statistics		Family of Related Emotions
	N (%) ^1^	L (%) ^2^	
**Anger**	312 (4.15)	9910 (5.67)	irritation, impatience, negative surprise, disappointment, bitterness, anger, irony, sarcasm, rage
*Examples: “The bubbles are still inactive—As they’ve been for five days;” “2. I’m a serious guy and i don’t have time for these divagations: (“Are you kidding me?; “I don’t believe it, you guys are in such a mess it’s unbelievable.”*			
**Fear**	102 (1.35)	3659 (2.09)	uncertainty, fear, worry, confusion, anxiety, panic
*Examples: “Pesel necessary:-O;” “GDPR”; “whoops… something’s gone wrong”*			
**Happiness**	761 (10.12)	21,521 (12.32)	interest, satisfaction, positive surprise, excitement, gratitude, hope, happiness, amusement
*Examples: “Well that’s great. Thank you sincerely);” “Concrete answer! Thank you! You helped me a lot!;” “Have a nice day XD”*			
**Neutral**	2269 (30.19)	90,817 (52.00)	not applicable
*Examples: “How long will the migration take?;” “I’m having trouble logging into the a;” “What are the internet access packages?”*			
**Sadness**	305 (4.05)	8741 (5.00)	resignation, bitterness, helplessness, regret, melancholy
*Examples: “I’ve been misled: (“I’ve written to you, but unfortunately no one is writing back;” “But it doesn’t work on my phone yet: (Why?)”*			

^1^ N (%), total number of fragments and their percentage in the sample; ^2^ L (%), total duration of fragments and their percentage in the sample.

**Table 4:** Effectiveness of automatic detection of affective states in voice channels.

No	Classifier Type	Voice Channel					
		*Accuracy*	SD ^1^	*Precision*	SD ^1^	*F1-Score*	SD ^1^
		[%]	[%]	[%]	[%]	[%]	[%]
1.	CNN	72.9	2.58	80.7	0.89	75.0	1.58
2.	kNN	70.0	1.00	67.8	1.64	67.2	1.64
3.	SVM	69.2	3.11	69.4	5.81	63.4	3.84

^1^ SD—Standard deviation.

**Table 5:** Effectiveness of automatic detection of affective states in text channels.

No	Classifier Type	Text Channel					
		*Accuracy*	SD ^1^	*Precision*	SD ^1^	*F1-Score*	SD ^1^
		[%]	[%]	[%]	[%]	[%]	[%]
1.	ANN	63.9	2.45	64.0	2.49	64.4	2.57
2.	DT	54.4	2.23	58.1	4.34	55.6	3.14
3.	kNN	55.5	2.14	62.1	4.98	56.1	3.45
4.	RFC	53.4	1.58	66.0	2.81	55.8	2.78
5.	SVM	49.2	1.54	57.7	4.57	49.1	4.18

^1^ SD—Standard deviation.

**Table 6:** Results of verification of the machine emotion detection process.

No	Classifier Type	Accuracy [%]	Precision [%]	F1-Score [%]
VOICE CHANNEL				
1.	CNN	67.5	80.3	74.5
2.	kNN	52.7	67.6	57.5
3.	SVM	62.4	63.9	62.2
TEXT CHANNEL				
1.	ANN	55.8	62.4	58.4
2.	DT	49.6	58.9	53.4
3.	kNN	55.3	57.2	55.7
4.	RFC	56.5	59.2	57.0
5.	SVM	65.9	58.5	61.7

## Data Availability

The research was conducted based on real talks obtained from a large Contact Center system.
